# A rare complication in adult undergoing chemotherapy for lung carcinoma: external jugular vein thrombophlebitis

**DOI:** 10.11604/pamj.2022.42.44.35263

**Published:** 2022-05-17

**Authors:** Yash Mohan Jakhotia, Avinash Parshuram Dhok

**Affiliations:** 1 Department of Radiodiagnosis, Narendra Kumar Prasadrao Salve Institute of Medical Sciences and Research Centre, Nagpur, Maharashtra, India

**Keywords:** External jugular vein, thrombophlebitis, ultrasonography, chemotherapy

## Image in medicine

A 40-year-old man who was diagnosed with a case of small cell lung carcinoma on chemotherapy (cisplatin and carboplatin) came with complaints of swelling and pain in the left neck and shoulder region for 5 days. On inspection, redness of skin was present in the neck region, and on palpation, it showed a local rise in temperature. The patient was further advised ultrasonography which revealed complete thrombosis of the external jugular vein on the left side with vessel wall thickening and surrounding inflammatory changes. The patient was started on anticoagulants further. After 6 months follow-up ultrasound sonography (USG) was done which revealed complete resolution of thrombus. Superficial vein thrombophlebitis is an extremely rare complication seen in patients having chemotherapy. Thrombosis in the external jugular vein is rare as compared to the internal jugular vein. Common etiologies of jugular vein thrombosis include trauma, central venous catheterization, infections, and extrinsic compression by tumors.

**Figure 1:**
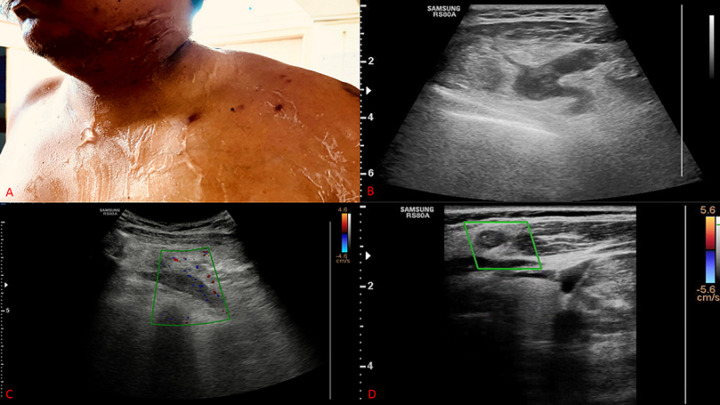
A) swelling and redness in neck region; B) B-mode ultrasonography image in longitudinal section showing complete thrombosis of external jugular vein; C) color Doppler image showing no flow in external jugular vein in longitudinal section; D) color Doppler image showing no flow in external jugular vein in transverse section

